# Ecological Threats and Cultural Systems: Epidemics and Natural Disasters Do Not Predict Collectivism

**DOI:** 10.1007/s12110-024-09480-8

**Published:** 2024-11-28

**Authors:** Soheil Shapouri, Yasaman Rafiee

**Affiliations:** 1 Department of Psychology, Lehigh University, 17 Memorial Dr. E, Bethlehem, PA 18015 USA; 2 Department of Psychological Sciences & Health, University of Strathclyde, Glasgow, UK

**Keywords:** Parasite stress, Pathogen prevalence, Natural disasters, Collectivism, Individualism

## Abstract

Considering the role of human interactions in infectious disease outbreaks and cooperation in mitigating natural disasters consequences, ecological threats to human survival have been among proposed drivers of collectivism. Utilizing established and novel measures of parasite stress and natural disasters, we investigated their association with collectivism in a large sample of countries (*N* = 188). Linear mixed-effect models indicated that after controlling for national wealth, neither natural disasters nor infectious disease can predict collectivism scores. Null results were consistent across different measures of threats, suggesting that previous findings can be attributed to small, non-representative samples of cultures. When universal patterns are a major concern, drawing conclusions based on small, nonrepresentative subsets of cultures risks promoting unreliable findings. Future cross-cultural research will benefit from data-driven exploratory methods to uncover factors previously unexamined in the theory-driven studies of collectivism.

**Supplementary Information:**

The online version contains supplementary material available at 10.1007/s12110-024-09480-8.

The proposition of parasite stress theory (PST) to explain the geographical variability of psychological traits (Fincher & Thornhill, 2012b) has resulted in a line of research that connects collectivism and its correlates to parasitic stress. Compared to other evolutionary and cross-cultural theories of collectivism, PST has received more attention and substantial empirical support (see Shapouri, 2023 for a review). In tandem, researchers have pointed to the phylogenetic and spatial non-independence of countries in cross-cultural comparisons to criticize PST research (e.g., Currie & Mace, 2012; Bromham et al., 2018). While the focus of critics of PST has largely revolved around statistical non-independence, the underrepresentation of less-developed countries in previous studies, the methods of measuring pathogenic stress, and the role of other ecological threats like natural disasters in driving collectivism have been relatively ignored. This manuscript aims to address these issues to advance further our understanding of the role ecological threats play in shaping cultural systems. We first provide a brief review of PST and then discuss methodological issues that need to be addressed.

## Parasite Stress Theory of Values and Sociality

Due to variations in pathogen prevalence across different regions and localized host immune adaptations, adaptive responses differ from place to place. For instance, the degree of assortative sociality, which includes limited dispersal, ethnocentrism, and xenophobia, varies as a function of the prevalence of pathogens in different parts of the world. Environments with high pathogen density exhibit more constrained dispersal, stronger ethnocentric views, and heightened xenophobia, all of which are indications of collectivism. Conversely, collectivist behaviors and cognitions may not be advantageous in ecologies with lower disease stress. Instead, individualism, which involves devaluing in-group assortative sociality is encouraged (Fincher & Thornhill, 2012a).

The seminal paper by Fincher et al. (2008) provided the first empirical support for connecting parasite stress and collectivism. This study demonstrated that the prevalence of infectious diseases predicts collectivism, even after controlling for confounding variables such as Gross Domestic Product (GDP), wealth distribution inequality, and population density. Following the publication of this landmark paper, the historical pathogen prevalence index (Murray & Schaller, 2010) has been used for cross-cultural studies (e.g., Gorodnichenko & Roland, 2017; Bastian et al., 2019), and the theoretical framework that PST provides has guided research in various domains of evolutionary psychology like women’s mate preferences (Clarkson et al., 2020), men’s beardedness (Pazhoohi & Kingstone, 2020), and personality traits (Mullett et al., 2020).

As the body of literature supporting PST grows, there has also been some criticism of its findings. However, most of these criticisms have centered around the issue of non-independence of units of analysis (Currie & Mace, 2012; Ross & Winterhalder, 2016; Horita & Takezawa, 2018; Bromham et al., 2018; Ma, 2020) while other methodological issues are not yet properly addressed in PST literature.

## External Validity

The issue of generalizability of psychological findings derived from WEIRD (Western, Educated, Industrialized, Rich, and Democratic) samples has gained widespread recognition and acknowledgment (Henrich et al., 2010). In cross-cultural evolutionary psychology, where the identification of universal patterns and locally specified adaptive solutions are common concerns, the importance of generalizability becomes even greater, not less (Apicella et al., 2020).

Researchers have relied on the best available measures of collectivism in PST research. Hofstede et al. (2001), Suh et al. (1998), Gelfand et al. (2004), and Minkov et al. (2017) are the most common measures used in individualism-collectivism research, but each of these measures suffers from methodological problems. Hofstede’s work on individualism-collectivism was based solely on surveys of IBM employees, with data collected over decades. His quantification of dimensions of national cultures, published in his books and on his website, rarely underwent rigorous peer review. While his work greatly facilitated comparative research, it faces theoretical inconsistencies (Oyserman et al., 2002) and psychometric problems (Brewer & Venaik, 2011). Suh et al. (1998) essentially used Hofstede’s individualism scores, supplemented by Harry Triandis’s expert ratings for nations not covered by Hofstede. Therefore, all criticisms of Hofstede’s work apply to Suh’s measure as well.

Subsequent studies on collectivism (e.g., Gelfand et al., 2004; Minkov et al., 2017) have attempted to address the methodological shortcomings of previous measures by, for example, using more representative samples. However, these measures still cover only about 60 nations. This limited coverage of global cultures applies to all the collectivism measures mentioned above, as they include at most about half of the countries in the world. A more severe problem is the bias of these measures toward including WEIRD countries and excluding less developed nations (Pelham et al., 2022).

Previous studies of PST can be used to illustrate the importance of a broader coverage of cultures in this line of research. Considering the higher frequency of the short allele of 5-HTTLRP in East Asians than in European populations, Chiao and Blizinsky (2010) found a significant and strong relationship between collectivism and the short allele of 5-HTTLRP in a sample of 29 nations. But when other researchers (Minkov et al., 2015) increased the sample size to more than 40 and included sub-Saharan African countries, the correlation between S-allele prevalence and collectivism turned insignificant. This suggests that a significant, strong relationship found in a small sample might disappear in a larger, more diverse sample. It is important to note that the small sample size problem equally applies to many studies that support PST (e.g., Tybur et al., 2016, *N* = 30), as well as studies that question its findings (e.g., Bromham et al., 2018, *N* = 50). These examples underscore the need to reevaluate, whenever possible, the conclusions of previous studies that were based on small, non-representative samples. Conducting such reevaluations with a larger and more representative sample is the first and overarching goal of the current study.

## Measures of Pathogenic Stress

The historical prevalence of infectious diseases (Murray & Schaller, 2010) remains the most common tool for multi-national studies of PST. This numerical index, derived from epidemiological atlases, is constructed based on the prevalence of nine infectious diseases: leishmaniasis, schistosomes, trypanosomes, leprosy, malaria, typhus, filariae, dengue, tuberculosis. These diseases were selected primarily due to the availability of worldwide records and their significant burden. However, as recent epidemics and pandemics like Ebola (Kim et al., 2016), swine flu (Hamamura & Park (2010), and COVID-19 (Na et al., 2021) have shown, other infections can also influence collectivism. The exclusion of some less deadly or more dangerous pathogens may lead to an underestimation or overestimation of the strength of the relationship between pathogens and collectivism.

Various sources that provide data on the prevalence of infectious diseases, each with its own advantages and disadvantages, have been used in this line of research. While the historical prevalence index offers a free and accessible tool, its coverage of infectious diseases is limited. In contrast, The Global Infectious Diseases and Epidemiology Online Network (GIDEON) provides valuable epidemiological data, historical records, and global disease status; however, it requires a subscription. A tool that is both free, and accessible, and offers a more comprehensive coverage of infectious outbreaks than historical prevalence index, would greatly facilitate research. Introducing such a tool is the second aim of the current study.

## Controlling for Other Ecological Threats: Natural Disasters

All prominent theories of collectivism, modernization theory, rice vs. wheat theory, climato-economic theory, and parasite stress theory attribute collectivism to different ecological factors (Shapouri, 2023). Hence, as a theory-driven field, cross-cultural research of collectivism has focused on a handful of variables: economic factors, subsistence strategies, the harshness of the climate and its interaction with economic resources, and the prevalence of infectious diseases. Natural disasters have not been the main focus as no specific theory directly attributes collectivism to natural disasters.

Similar to infectious outbreaks, natural disasters are direct threats to human survival that are experienced collectively, and cultural practices might make people less or more vulnerable to natural hazards. A few studies have investigated the role of natural disasters in driving collectivism, but they have produced mixed findings.

On the one hand, a few studies suggest natural disasters might increase collectivism or reduce individualism. A longitudinal study in the United States (Grossmann & Varnum, 2015) shows that disaster frequency is followed by more individualistic child-naming practices. On a worldwide stage, a study of 33 nations (Gelfand et al., 2011) found that tightness, the overall strength of social norms and tolerance of deviance, has a strong positive correlation with disasters such as floods, tropical cyclones, and droughts. On the other hand, in a study of about a hundred countries, Oishi and Komiya (2017) found that natural disaster risk predicts collectivism but not after controlling for environmental and economic factors like historical pathogen prevalence and national wealth. While the natural disaster risk index used in this study is based on the World Risk Index of 2013, their pathogen prevalence comes from Fincher and Thornhill’s study (2021a) which used WHO’s 2002 infectious diseases data. In other words, natural disasters and infectious diseases were measured at different time points. The World Risk Index is calculated based on vulnerability to earthquakes, storms, floods, droughts, and sea level rise, and excludes other events such as extreme temperature or insect infestation. Moreover, while infectious disease data measures the extent to which people are exposed to this threat, the World Risk Index takes many social and economic indicators into account, and as its creators put it, “shows that the vulnerability of a society or a country is not the same as exposure to natural hazards.” (Birkmann & Welle, 2015). So, while Oishi and Komiya’s study is a great contribution to the field, it has some methodological problems. The severity of natural disasters and infectious diseases were measured by completely different methods, they were measured at different time points, and some natural disasters, such as cold and heat waves, were not included. It is not clear whether their findings can be replicated if natural disasters and epidemics are measured with the same operational definition, measured at the same time, and without excluding any common natural disaster. Replication of their findings was the third goal of this study.

## Current Study

The ongoing emergence of infectious outbreaks exerts adaptive pressure on humanity, and the COVID-19 pandemic has reignited interest in research on the role of parasitic stress on collectivist behaviors. Moreover, the rise of climate change-related events requires a better understating of their large-scale psychological impacts. Through methodological refinement, this study seeks to contribute to the current body of literature by reexamining the effects of pathogenic stress and natural disasters on an important aspect of national cultures. By including more countries than previous studies, introducing a novel measure of pathogenic stress that is freely and publicly accessible, and conducting a parallel comparison of epidemics and natural disasters, our objective is to replicate prior findings and assess the robustness of the effects of large-scale ecological threats on individualism-collectivism. Moreover, considering the importance of transparency and replication in cross-cultural research (Damian et al., 2022b), we provide all the data and codes.

### Methods

We report on the data collection and preprocessing, data transformation, measures of the study, and data analysis. This was a cross-national, comparative study using archival data, with national wealth, natural disasters, and epidemics as predictors, and collectivism as the outcome.

### Materials

#### Ecological Threats

The Emergency Events Dataset (EM-DAT) (https://www.emdat.be/) was used to calculate the number of epidemics and natural disasters in each country along with the number of deaths caused by these threats that have happened from 1900 to 2000. EM-DAT is a disaster loss dataset developed and maintained by the Center for Research on the Epidemiology of Disasters (CRED). This dataset contains information related to every disaster and epidemic that has happened since 1900 if they met at least one of the following criteria: 10 or more people reported killed; hundred or more people reported affected; declaration of a state of emergency; or call for international assistance (https://public.emdat.be/about). Since CRED classifies epidemics as natural disasters, the records of these two threats were separated. Then the number and causalities of epidemics and natural disasters were aggregated for each country. EM-DAT aggregates records for countries that were part of the Soviet Union and Yugoslavia before their dissolution. In such cases, casualties of successor states are estimated based on their populations (see the ESM for details). To scale the casualties to the size of the population, the aggregated number of deaths for each country was divided by the average population between 1960 and 2000 retrieved from the World Bank Data (Taiwan population is not reported by the World Bank so we used macrotrends.net records).

A comparison between our measure of pathogenic stress and the historical pathogen prevalence index revealed that our measure includes some of the prevalent diseases covered by historical pathogen prevalence index (i.e., leishmaniasis, typhus, and dengue). However, other infectious diseases included in the historical pathogen prevalence index, such as schistosomiasis, which did not reach epidemic levels in the twentieth century, are not covered by our measure. Instead, our measure includes more prevalent diseases like cholera, yellow fever, and meningococcal. More importantly, our measure also incorporates diseases such as Ebola, SARS, and influenza, which studies suggest may influence collectivist behaviors. Our measure of natural disasters included a wide range of biological, climatological, geophysical, hydrological, and meteorological events, such as earthquakes, volcanic activity, droughts, and wildfires.

#### National Wealth

The GDP per capita (current US$) figures for countries in the year 2000 were obtained from the World Bank Data (https://data.worldbank.org/). The GDPs of 2000 were missing for six countries in World Bank data. In such cases, we used either another year as an estimate of the 2000 GDP or the GDP of 2000 from another source instead of the World Bank (for details see the ESM).

#### Collectivism

The Global Collectivism Index (GCI) was used as the primary outcome. GCI is the most recent measure of collectivism that shows good psychometric properties, and it has moderate to strong correlations with other measures of collectivism, including Hofstede (0.65), Minkov et al. (0.88), GLOBE (0.68); and Suh et al. (0.69) (Pelham et al., 2022). It includes the scores for 188 countries and regions of the world and contains more non-WEIRD countries than any other measures of individualism-collectivism (Pelham et al., 2022). Utilizing this measure enabled us to achieve broader coverage of countries compared to previous studies. Unlike previous measures of collectivism, the GCI is a composite index that incorporates both objective measures (e.g., family size) and nationally representative self-report surveys (e.g., religiosity). Given its broader cultural coverage, strong psychometric properties, and reliable measurements, the GCI is an excellent tool for cross-national studies of collectivism.

#### World Regions

The majority of researchers believe that data points created by shared history, geography, and proximity in cross-cultural research are not independent (Claessens et al., 2023; Bromham et al., 2018; for an opposite view see Thornhill & Fincher, 2013) but independence of observations is the assumption of many statistical procedures. To address the non-independence of countries we included world regions as a control. Following previous studies (e.g., Hruschka & Henrich, 2013), world regions were classified into East Asia and Pacific, Europe and Central Asia, Latin America and the Caribbean, Middle East and North Africa, North America, South Asia, and Sub-Saharan Africa according to the World Bank (2024).

#### Historical Pathogen Prevalence Index

To check whether our results are better explained by greater coverage of cultures or the new measure of pathogenic stress we also included historical pathogen prevalence index (Murray & Schaller, 2010). This index has two 9-item and 7-item versions. We used scores of 7-item version as it covers more countries. Historical pathogen prevalence index does not report scores for Montenegro and Palestine so our sample size for this analysis was 186. The data can be found in Supplementary Data S2 (10.17605/OSF.IO/58DJP).

### Data Analysis

Two sets of analyses were conducted: bivariate correlation and mixed-effects regression. To make the results comparable to similar studies (e.g., Fincher et al., 2008; Gelfand et al., 2011, Tybur et al., 2016), we have reported correlation coefficients and to control for relevant factors, we used regression. As mixed-effect modeling with geographical entities is the most common practice for controlling non-independence in this line of research (e.g., Hruschka, & Henrich, 2013; Zhang, 2018; Pasin et al., 2024), we included regions as a random effect in all models.

We first calculated zero-order Pearson correlation coefficients. But visualization of data indicated right-skewness and existence of potential outliers for all predictors. As Pearson coefficient is sensitive to outliers (Kim et al., 2015), it has been suggested to use Spearman in case of heavy-tailed distributions (De Winter et al., 2016) and presence of outliers (Kim et al., 2015). Therefore, to make the results comparable to previous studies we have reported Pearson correlation coefficients, but we have also reported Spearman coefficients that are more reliable considering the distributions of variables (see Results).

As variables were in different units and ranges, we used rank-based inverse normal transformation on all predictors. We then constructed three random-intercept models using restricted maximum likelihood (REML): the first model included GDP, the number of epidemics, and disasters as fixed effects; the second accounted for GDP and deaths from epidemics and disasters; the third focused on GDP and mortality rates from these events. World regions were included in all models as random intercepts.

Statistical analysis was performed using R software version 4.3.2 and R Studio version 2023.12.1 + 402. The packages used included “Hmisc” for the correlation matrix, “RNOmni” for inverse-rank normal transformation, “lmerTest” for mixed-effect modeling, “car” for calculating variance inflation factors (VIF), and “influence.ME” for assessing influential observations based on Cook’s distance. The codes for statistical analysis can be found in supplementary Data Analysis S3 (10.17605/OSF.IO/58DJP).

### Results

As shown in Table 1, all predictors exhibit significant skewness. Therefore, when interpreting the results in Table 2, the Spearman correlation coefficients should be considered more reliable. These bivariate correlations indicate a significant relationship between collectivism and epidemics, but not disasters except disaster mortality. However, due to potential confounding factors (particularly national wealth and non-independence), these correlations do not permit a definitive conclusion. They are presented primarily for analytical purposes and to facilitate comparison with previous studies.

**Table 1:** Descriptive statistics

	Mean	Median	SD
GDP	7272.27	1901.61	11410.11
Number of epidemics	3.97	2	6.28
Number of disasters	36.22	13.5	69.88
Deaths by epidemics	50577.77	62.5	364,274
Deaths by disasters	114628.3	476	875335.5
Epidemics mortality	0.001	0.0000064	0.003
Disasters mortality	0.003	0.00009	0.02

**Table 2:** Zero-order correlation coefficients (*n* = 188)

	Global Collectivism Index	
	Pearson coefficients	Spearman coefficients
Number of epidemics	0.41***	0.54***
Number of disasters	−0.10	0.00
Deaths caused by epidemics	−0.01	0.42***
Deaths caused by disasters	0.01	0.11
Epidemics mortality	−0.01	0.43***
Disasters mortality	0.07	0.17*
GDP	−0.67***	−0.80***

***p <.001, **p <.01, *p <.05

To further investigate the relationships between ecological threats and collectivism three random-intercept mixed-effect models were run and their results are presented in Table 3. In all three models, residuals were normally distributed, there was not any significant multicollinearity (all VIFs below 1.69), and removing influential observations based on Cook’s distance did not change the significance of any predictors.

**Table 3:** Mixed-effect models (*N* = 188)

Predictor	β (SE)	t(df)	95% CI
**Model 1**			
GDP ***	−0.52 (0.04)	−12.97 (183.52)	[− 0.60, − 0.45]
Number of epidemics	0.015 (0.04)	0.341 (181.43)	[− 0.07, 0.10]
Number of disasters	−0.03 (0.03)	−0.75 (183.48)	[− 0.10, 0.04]
Marginal R² / Conditional R² 0.54 / 0.72 **Model 2**			
GDP ***	−0.55 (0.04)	−13.58 (183.49)	[− 0.64, − 0.48]
Deaths by epidemics	−0.06 (0.04)	−1.4 (182.16)	[− 0.13, 0.02]
Deaths by disasters	0.01 (0.03)	0.18 (181.12)	[− 0.06, 0.07]
Marginal R² / Conditional R² 0.53 / 0.72 **Model 3**			
GDP ***	−0.54 (0.04)	−12.92 (183.91)	[− 0.618, − 0.455]
Epidemics mortality	−0.05 (0.04)	−1.28 (181.80)	[− 0.12, 0.02]
Disasters mortality	0.05 (0.03)	1.547 (181.21)	[− 0.01, 0.11]
Marginal R² / Conditional R² 0.52 / 0.72			

***p <.001

As Table 3 shows in all three cases, only GDP was the significant predictor of collectivism. To further evaluate models, we compared reduced models (i.e., dropping one variable at a time) with full models (i.e., including all predictors) using ANOVA. All evaluations indicated that models with epidemics or disasters were not significantly different from models with only GDP and regions (see Data Analysis S3 for details).

To assure that the null results above cannot be attributed to any potential problem with our new parasite stress measures, we also checked the association between historical pathogen prevalence index and collectivism controlling for GDP and including region as random intercepts. The results showed only a significant association for GDP (β *=* −0.32, *SE* = 0.02, *t*(182.48) = − 14.28, *p* <.001, 95% CI [− 0.36, − 0.28]) but not pathogen prevalence (β *=* 0.09, *SE* = 0.07, *t*(182.98) = 1.42, *p* =.16, 95% CI [− 0.03. 0.23]).

Finally, since we introduced a new measure of pathogenic stress, we investigated the relationship between our measure of pathogenic stress and historical pathogen prevalence index. The number of epidemics (*ρ* (186) = 0.57, *p* <.001), deaths caused by epidemics (*ρ* (186) = 0.40, *p* <.001), and mortality from epidemics (*ρ* (186) = 0.34, *p* <.001) were all significantly positively correlated with historical pathogen prevalence index.

## Discussion

While cross-cultural researchers continue looking for drivers of national cultures, ecological factors remain the most important variables of interest in explaining geographical variations in individualism-collectivism. With the largest sample size possible, we investigated the effects of two ecological threats to human survival on collectivism and the results of mixed-effect modeling did not support the parasite stress theory or the effect of natural disasters on collectivism.

Regarding natural disasters, our measure of natural disasters included a wider range of events like extreme temperature not covered by World Risk Index, but our findings are in agreement with Oishi and Komiya (2017), reinforcing the conclusion that these events do not exert an influence on collectivism. One of the earliest studies of this issue (Gelfand et al., 2011) has only reported correlations without controlling confounds so our findings do not contradict any cross-cultural comparisons. It is important to note that based on our own findings we cannot refute the possible role of disasters in shaping other social behaviors besides collectivism. Some studies, for example, suggest an increase in prosocial behavior following natural disasters (Maki et al., 2019) although these effects might be attributed to a general tendency toward helping people in distress or mitigating property damage caused by disasters (Vardy & Atkinson, 2019). But property damage could not be a major concern for hunter-gathers with very few possessions, and altruistic behavior is not restricted to the aftermath of natural disasters. So, while natural disasters might promote prosocial behavior, they do not increase collectivism.

Considering parasite stress theory, in the absence of any systematic review or meta-analysis it is difficult to make a firm conclusion, but it seems the literature is mixed and inconclusive at this point. While some studies support the link between infectious disease and collectivism (e.g., Cashdan & Steele, 2013), some have yielded mixed results (e.g., Culpepper, 2019), yet others have found no significant relationship (Horita & Takezawa, 2018). These discrepancies may stem from different issues and in conjunction with our results might have different explanations and implications.

First, although independent variables are the same across different studies of parasite stress (i.e., typically pathogen prevalence measured by historical pathogen prevalence index), but dependent variables vary, and this variation in outcomes might explain some contradictory findings. Our study in combination with others who did not find any association between infections at individual-level (Fischer, 2021) and country-level human values (Ma, 2020) question the direct, straightforward link between infections and collectivism or its correlates. It is still possible that parasitic stress affects some aspects of group-oriented psychology at least in specific regions of the world, like freedom of speech in wealthy nations (Kusano & Kemmelmeier, 2018). Moreover, it is possible that while parasite stress does not directly affect collectivism, in interaction with subsistence strategies it mediates the link between climate harshness and collectivism (Van de Vliert, 2020) but these findings need to be replicated in more representative samples as well.

Second, sample characteristics and size matter. A study of 30 nations by Tybur and his colleagues (2016) indicated more relevance of parasite stress to intragroup norms than intergroup motivations but another study of about 80 countries (Zhang, 2018) found the opposite: no effect of infectious disease on in-group trust but a curvilinear relationship with out-group trust. This suggests that findings from a subset of nations might not be extended to a more extensive sample. Drawing conclusions about human universals from a convenience sample of a limited number of countries is therefore precarious. Our findings indicate that the link between parasite stress and collectivism cannot be universally applied across all cultures.

This study is not without limitations. 56% of cross-national studies use multilevel modeling (Damian et al., 2022a) and since our main goal was replication of previous findings, we used mixed-effect modeling as the most common method for controlling spatial and cultural non-independence. However, multilevel modeling with regions as categorical random effects has its own methodological issues. There is no consensus on how to group countries together to better reflect historical processes and spatial proximity that result in non-independence. We used World Bank classification as a common and convenient method but classifications by World Bank or United Nations are based on geographical proximity as well as geopolitical considerations. Other grouping methods such as those proposed by GLOBE project (House et al., 2004) which take cultural similarities into account might be better for the investigation of national cultures. Moreover, using categorical random effects as a proxy of continuous spatial proximity and cultural similarities can affect type I and type II error rates and precision of the model (Silk et al., 2020). It is possible that our crude treatment of non-independence might have masked the effect of epidemics or exaggerated the role of GDP. In the absence of comprehensive robust phylogenies of all cultures some researchers have proposed using linguistic distances as a proxy of phylogenetic non-independence (i.e., Bromham et al., 2018). Including linguistic and spatial proximity as a covariance matrix in Bayesian phylogenetic models to control for non-independence can reduce type I error rates while maintaining statistical power (Claessens et al., 2023). While these methods might be more suitable for some cross-national studies, the fact that we did not employ them in our study should not be a major concern. The primary focus of these methods is typically on reducing type I error, but since we did not find any significant relationship, the possibility of a spurious correlation is not relevant. Moreover, while phylogenetic methods do reduce type I error, commonly used methods still possess acceptable statistical power, even under conditions of strong spatial autocorrelation, provided that the correlation between the predictor and outcome variables is large (Claessens et al., 2023). Given the correlation between epidemics and collectivism, along with our large sample size, it is highly unlikely that we missed a true correlation.

Our study was limited to a few variables, national wealth as the strongest predictor of collectivism, parasite stress and natural disasters as our primary variables, and we focused on replication of previous findings with a larger, more representative sample. But future research does not have to be limited to a few variables considered important by theories. With the advent of big data, data mining, and machine learning algorithms, it is possible to investigate hundreds, even thousands of variables simultaneously. For example, random forests can automatically model linear and non-linear relationships with complex interactions, and they have been recently adjusted for mixed-effect modeling (Hajjem et al., 2014). These methods allow for variable selection based on empirical data. This data-driven approach can relax the constraints of theory-based research and possibly result in discovering variables that might have been overlooked by theories (Jack et al., 2018) that have come short in explaining geographical variations of cultures. For now, it is safe to assume that macro-cultural dimensions like collectivism are resistant to change even when they face several epidemics or a global pandemic (Pasin et al., 2024).

## Electronic Supplementary Material

Below is the link to the electronic supplementary material.

Supplementary Material 1

## Data Availability

See the ESM for Data Compilation (S1). The data and codes are available as open data via the Open Science Framework data repository: 10.17605/OSF.IO/58DJP.
