# Supplementary material for: Ecological Threats and Cultural Systems: Epidemics and Natural Disasters Do Not Predict Collectivism
Source: Hum Nat. 2024 Nov 28;35(4):382–96. doi: 10.1007/s12110-024-09480-8 (PMC11835910; doi:10.1007/s12110-024-09480-8)
Supplement: Supplementary file 1 — Supplementary Material 1 [file 12110_2024_9480_MOESM1_ESM.docx]

Electronic Supplementary Material

for

Ecological Threats and Cultural Systems: Epidemics and Natural Disasters Do Not Predict Collectivism

Soheil Shapouri^1^, Yasaman Rafiee^2^

^1^Department of Psychology, Lehigh University, Bethlehem, PA, USA

^2^Department of Psychological Sciences & Health, University of Strathclyde, Glasgow, UK

Correspondence: [sos523@lehigh.edu](mailto:sos523@lehigh.edu)

*Human Nature* 35(4), 2024, OSF doi: 10.1007/s12110-024-09480-8

**Data Compilation (S1)**

**Ecological Threats – Casualties**

In two cases where a county was dissolved, casualties of epidemics and natural disasters are estimated as follows:

**Soviet Union**: First, the proportions of populations of countries that were part of the Soviet Union were calculated based on the [populations in 1979](https://en.wikipedia.org/wiki/Demographics_of_the_Soviet_Union#Population). Then epidemics and natural disaster data were estimated based on proportions. For example, consider the disaster 1921-9001-SUN in EM-DAT, a drought in the Soviet Union that killed 1,200,000 and its location reported is Kazakhstan, Russian Federation, Ukraine.

The populations of these three countries in 1979 were as follows: Kazakhstan: 14,685,000; the Russian Federation: 137,551,000; Ukraine: 49,755,000.


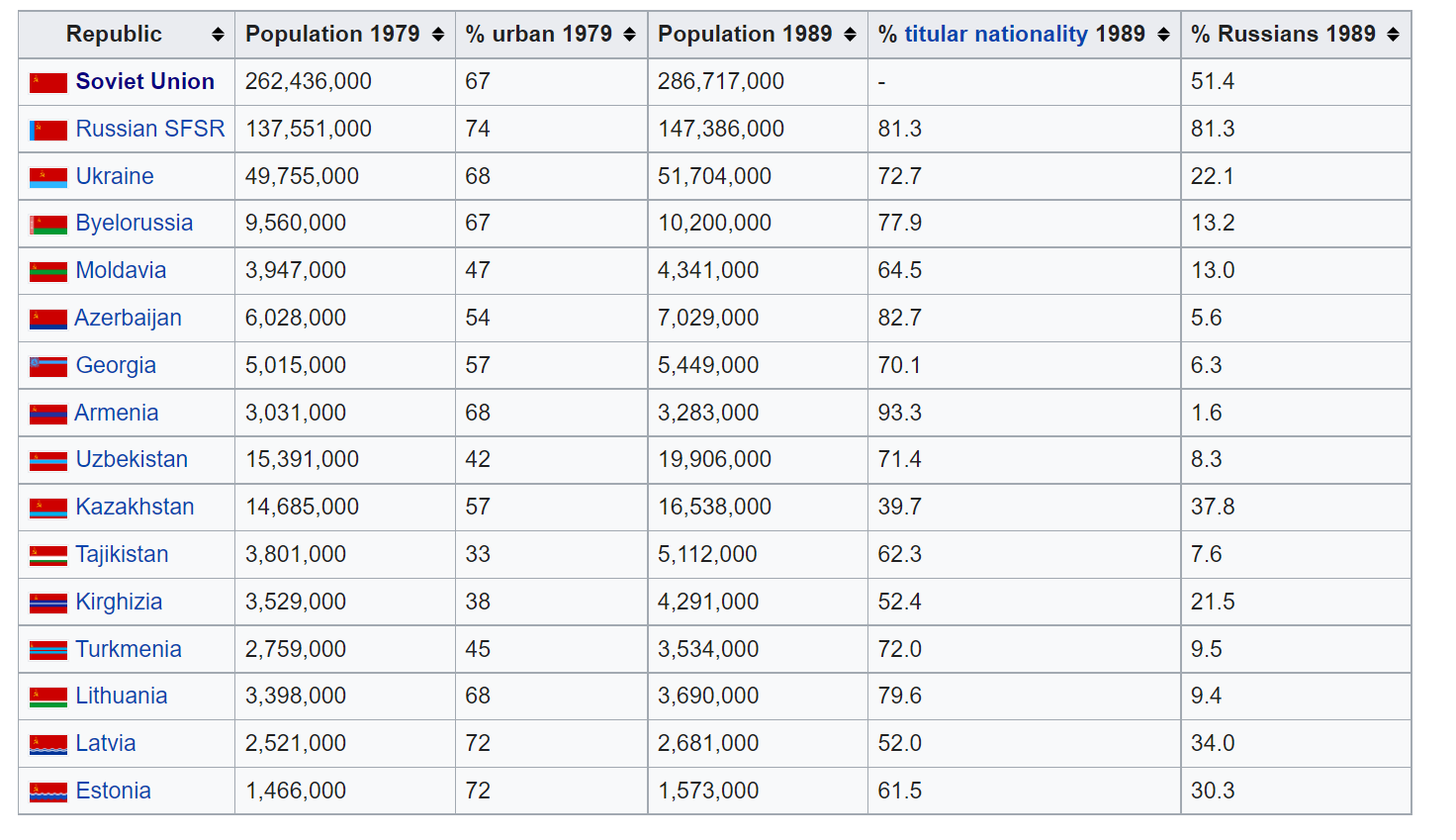


Source: [https://en.wikipedia.org/wiki/Demographics_of_the_Soviet_Union#Population](https://en.wikipedia.org/wiki/Demographics_of_the_Soviet_Union#Population)

Since the total population of the three countries involved in this disaster was 201,991,000, we estimated the total number of deaths for this event in Kazakhstan as 87,241.

**Yugoslavia**: A similar procedure was used for estimating epidemics and natural disaster statistics in the Socialist Federal Republic of Yugoslavia and the population proportions were taken from [Wikipedia.](https://en.wikipedia.org/wiki/Demographics_of_the_Socialist_Federal_Republic_of_Yugoslavia)


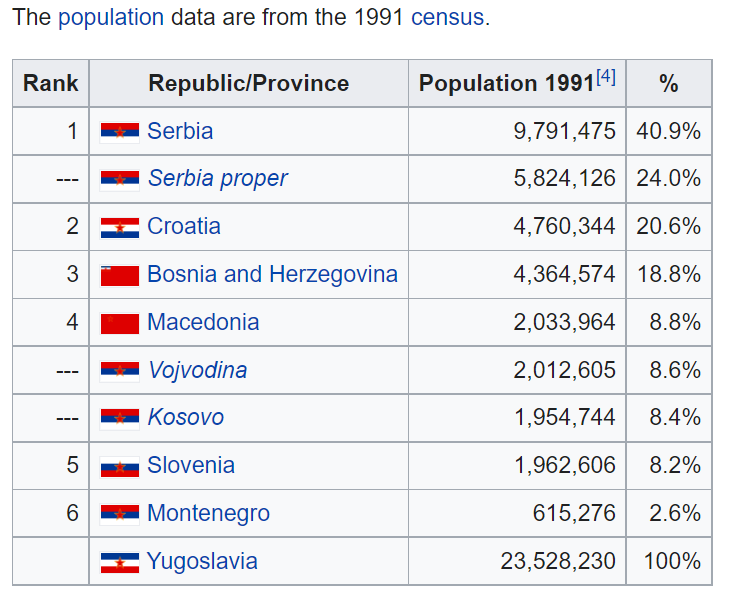


Source: [https://en.wikipedia.org/wiki/Demographics_of_the_Socialist_Federal_Republic_of_Yugoslavia#Republics_by_population](https://en.wikipedia.org/wiki/Demographics_of_the_Socialist_Federal_Republic_of_Yugoslavia#Republics_by_population)

Since Wikipedia pages may be edited, a screenshot of tables used is reprinted here.

**GDP per Capita**

In six cases, the GDP data for the year 2000 was not available in the World Bank records. We used the GDP figures from the closest available year from World Bank data for Afghanistan, Guam, Somalia, and the Virgin Islands (U.S.).

| Country | Year |
| --- | --- |
| Afghanistan | 2002 |
| Gaum | 2002 |
| Somalia | 1990 |
| Virgin Islands (U.S.) | 2002 |

For North Korea, we got the 2000 GDP from Our World in Data website (<https://ourworldindata.org/grapher/gdp-maddison-project-database?tab=chart&country=PRK>) then changed 2011 US $ to current US $ (2023) using <https://www.officialdata.org/>.

We got the 2000 GDP per capita for Taiwan from the International Monetary Fund: <https://www.imf.org/external/datamapper/NGDPDPC@WEO/ADVEC/WEOWORLD/TWN/CHN>
